# Development of a Framework and the Content for a Psychoeducational Internet-Delivered Intervention for Women after Treatment for Gynecological Cancer

**DOI:** 10.3390/nursrep11030061

**Published:** 2021-08-12

**Authors:** Ragnhild Johanne Tveit Sekse, Tine Nordgreen, Eivind Flobak, Morten Lystrup, Espen Braathen, Henrica M. J. Werner

**Affiliations:** 1 Department of Obstetrics and Gynaecology, Haukeland University Hospital, 5053 Bergen, Norway; 2 Department of Clinical Science, University of Bergen, 5009 Bergen, Norway; 3 Department of Clinical Psychology, Faculty of Psychology, University of Bergen, 5009 Bergen, Norway; 4 Faculty of Health Studies, VID Specialized University, 5009 Bergen, Norway; Morten.lystrup@vid.no (M.L.); Espen.braathen@vid.no (E.B.); 5 Division of Psychiatry, Haukeland University Hospital, 5009 Bergen, Norway; tine.nordgreen@helse-bergen.no; 6 Department of Global Public Health and Primary Care, Faculty of Medicine, University of Bergen, 5018 Bergen, Norway; 7 Department of Information Science and Media Studies, University of Bergen, 5007 Bergen, Norway; Eivind.flobak@uib.no; 8 Department of Obstetrics and Gynecology, Maastricht University Medical Center, 6202 AZ Maastricht, The Netherlands; erica.werner@mumc.nl; 9 GROW—School for Oncology and Developmental Biology, Maastricht University Medical Center, 6202 AZ Maastricht, The Netherlands

**Keywords:** Internet-delivered intervention, psychoeducational intervention, gynecological cancer, rehabilitation, cancer survivorship, user involvement, complex intervention

## Abstract

The number of women treated for gynecological cancer is increasing. At the same time, the duration of in-patient hospitalization has decreased, and follow-up with its primary focus on early recognition of recurrence does not meet all patients’ needs. One method of follow-up may be digital intervention. This study describes the development of a psychoeducational Internet-delivered intervention targeting women’s psychosocial needs during the follow-up period after treatment for gynecological cancer. The project consisted of three phases following the UK Medical Research Council Framework guidelines for the development of complex interventions. Phase one identified the evidence in the field, phase two identified the relevant theoretical framework, and phase three included a two-year work process including focus group interviews and think aloud interviews with users. Through the steps of literature review, theoretical framework, and an iterative development process with users and other stakeholders, a six-week program was developed. The program included psychoeducational information, multimedia, exercises, and weekly telephone follow-up with a dedicated nurse. This Internet-delivered intervention can be a novel method for addressing the gap in the provision of follow-up for women after treatment for gynecological cancer.

## 1. Introduction

The number of women diagnosed and treated for gynecological cancer is increasing. At the same time, in-patient hospitalization time has decreased and follow-up, with its primary focus on early recognition of recurrence, does not meet all patients’ needs [1,2].

The majority of cancer survivors do not regain their previous level of health and psychosocial function [3,4,5]. Gynecological cancer survivors specifically face broad-ranged challenges concerning their physical, psychological, social, and existential well-being for years after completion of treatment [4,6]. Menopausal symptoms, lymphedema, challenges related to sexual health, womanhood, and infertility issues, fear of recurrence, anxiety, and depression are commonly reported [7,8,9,10]. Moreover, women have a need for additional information from and dialogue with health care professionals regarding the process of coping with changes and late effects [9,11] beyond routine care and follow-up.

A few psychosocial interventions in the field of cancer rehabilitation have been developed to fill this gap [12,13,14,15]. A review focusing on the most promising intervention strategies addressing health-related quality of life (QoL) concerns in gynecological cancer survivors [12] concluded that information alone seemed largely unable to provide clinically meaningful benefits, while psychosocial interventions provided some positive effects. Another systematic review [13] on the effectiveness of psychoeducational interventions for women treated for gynecological cancer confirmed that such interventions appeared to improve patients’ sexual functioning, reducing distress, anxiety, and depression. The review suggested that an effective design should be theory-based, incorporating information provision, behavioral therapy, and psychological support.

The past decade has seen an increase in the interest for Internet-delivered interventions that provide health information and psychosocial support in cancer follow-up [16,17]. Several reviews show that Internet-delivered interventions have the potential to add great value to the participants’ educational process and that such interventions, in most circumstances, are well-received [18,19,20]. Internet-delivered interventions have the advantage of being convenient, cost-effective, anonymous, interactive, easily updated and can provide links to other resources [21,22,23]. A meta-review [24] of Internet-delivered interventions for patients with cancer showed positive effects on perceived support, knowledge, and information competence. However, the review showed an inconsistent effect on QoL and psychological well-being [24]. Nevertheless, few Internet-delivered interventions are related to follow-up after gynecological cancer.

Summing up, the follow-up, with its primary focus on early recognition of recurrence, does not meet all patients’ needs and is subject to criticism. Additional approaches should be considered, much more so in an era of decreasing patient contact and increasing incidences of gynecological cancer cases. As cancer survivors live longer, often with long-term side effects and challenges, adapting to life after the illness and accepting a new life situation are of paramount importance. One scalable method to include psychoeducational interventions in the follow-up is Internet-delivered interventions.

The main aim of the study is to describe the development of a psychoeducational Internet-delivered intervention targeting the women’s psychosocial needs during the follow-up period after treatment for gynecological cancer.

## 2. Design and Methods

**Figure 1:**
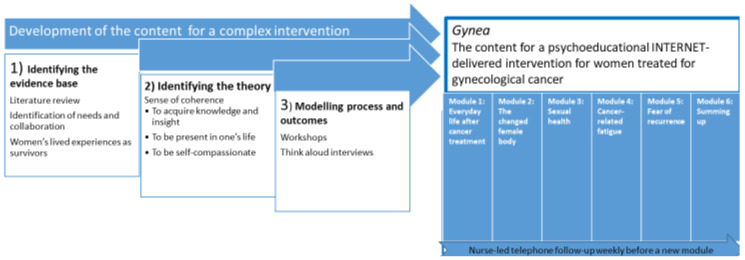
A model of the developmental process.

We used a development approach consistent with the framework recommended for developing complex interventions proposed by the UK Medical Research Council (MRC) [25,26]. Three stages in this developing process are elaborated: (1) identification of the evidence, (2) identification of a relevant theorical framework, and (3) modelling process and outcomes [25,26] (Figure 1).

Identification of the evidence (1) encompasses an integrative review, as well as elaborating on the available qualitative studies on women’s lived experiences as survivors after gynecological cancer.

Identification of the theoretical framework (2) was performed in close collaboration with the user group’s needs and with several stakeholders. This framework describes three pillars with Antonovsky’s sense of coherence [27,28] as an overall model.

Modelling process with outcomes (3) was an iterative two-year process with users and other stakeholders. The user representatives were recruited through the board of the Gynecological Cancer Patient Association. The inclusion criteria were as follows: women aged > 18 years, Norwegian-speaking, completed primary treatment for gynecological cancer. Five women volunteered and were active in workshops throughout the two-year period, and five additional women recruited through snowballing (total *n* = 10) participated in evaluation sessions of the program.

Written information about the study was given to all the users, and written informed consent was obtained from all the participants. They were all informed that participating in the study was voluntary and they could withdraw from the study at any time without providing any reason. Audio files and transcripts of the workshops and think aloud evaluations were stored at the research server at the hospital for research purposes. The confidentiality of the participants was assured and the information that could identify anyone was omitted from the material.

The study conformed to the principles outlined in the Declaration of Helsinki. Moreover, the study was exempted from ethical approval, confirmed by the Medical Research Ethics Committee (REK) (2018/1356), and the authors reported to the data protection officer at Haukeland University Hospital (2018/11263).

## 3. Development Process

### 3.1. Identification of the Evidence

A broad search for existing evidence was conducted in line with the MRC guidance [26], collecting evidence from an integrative review and data from qualitative studies.

#### Literature Review

An integrative review of Nordic women’s experiences and quality of life after treatment for various forms of gynecological cancer (55 studies; > 3000 participants) was performed at the same time as preparing for the current study [6]. The review revealed that for years after treatment, women had to deal with fundamental changes and challenges concerning their physical, mental, and psychosocial well-being. *Physical well-being in a changed body* reported changes including menopausal symptoms, a changed sexual life, complications with the bowels, urinary tract, lymphedema, and pain. *Mental well-being* dealt with questioned womanliness, the experience of revitalized values in life as well as fear of recurrence. *Psychosocial well-being and interaction* comprised the importance of having a partner or close person in the process of coming to terms with oneself after cancer. The review clarified the women’s needs for more information and follow-ups, as well as their search for strategies for coping with changes and late effects [6].

Encompassed in the current review, several studies dealt with the women’s *lived experiences* as survivors after gynecological cancer, a perspective the users asked for. The first-person perspective in terms of women’s lived experiences could make the content more authentic and attractive and integrate health information. This perspective is also recommended in the person-based approach to developing usable and engaging health-related interventions [29]. Moreover, a first-person perspective may help users identify with other women in similar situations, normalizing the changes and challenges in the aftermath and possibly reducing feelings of loneliness. As such, the first author’s previous studies with in-depth interviews with long-term survivors after gynecological cancer [30], as well as focus group interviews with women attending an education and counseling group intervention [31], were analyzed anew, aiming to use the experiences of the first-person perspective in the intervention’s content [29].

Moreover, we elaborated on three narratives from a typology that featured three different ways of coping with and processing cancer [32]. Additionally, the three narratives were attributed to different types of gynecological cancer, treatment modalities, side effects, age, and social context. Through diversifying the characters, we aimed to provide narratives that future users could easily identify with.

### 3.2. Identification of the Relevant Theorical Framework

Application of a relevant theoretical framework is central to a useful and successful intervention [26]. The conceptual framework of this intervention was based on three complementary theoretical perspectives with Antonovsky’s coping strategy “sense of coherence” as overall thinking [27,28]. Based on this overall thinking, we encompassed three central concepts, or pillars, to recur throughout the program: (1) To acquire knowledge and insight, (2) To be present in one’s life, and (3) To be self-compassionate.

To acquire knowledge and insight (1) encompasses relevant medical information that can yield insight in the illness and treatment-related changes and challenges. Information provides an increased sense of control and awareness, which in turn can support better coping in the aftermath of cancer treatment. Through both factual information and taking part in other patients’ experiences, new insights, understanding, as well as manageability are promoted [27]. Taking part in someone else’s thoughts, emotions, and challenges can help the individual to become more understanding of one’s own situation. Similarly, in this psychoeducational intervention, writing one’s own story of illness can help the individual to reconstruct the perception of one’s relationship to the world [33]( p. 3)

To be present in one’s life (2) implies two main perspectives: awareness and acceptance. Presence is a type of balanced awareness that neither resists, exaggerates, nor avoids any moment of one’s experience. Accepting a critical situation means both grieving over what is lost and being open for the opportunities in life here and now [34,35]. To facilitate this process of accepting one’s present life situation, mindfulness-based exercises can be helpful. Mindfulness promotes the ability to observe thoughts, emotions, bodily sensations, and the outside world without judging or trying to change anything [36]. Applying mindfulness to psychoeducational interventions, women may find the resources to accept their current situation and build resilience. Thus, they may be better equipped to handle rumination and fear of cancer recurrence (FCR).

To be self-compassionate (3) is the third concept of the program and presupposes the second one, mindful awareness. Self-compassion means to be considerate with oneself, comparable to the way one takes care of others [37] (p. 16). The self-compassion perspective and practice may give us flexibility and resilience in facing our human condition [38,39]. Practicing mindful self-compassion exercises may enhance the women’s resources in coping with suffering as cancer survivors.

### 3.3. Modelling Process and Outcomes

The MRC recommends an iterative development process prior to proceeding to clinical testing [26,40]. Collaboration with user representatives ensures the program meets the patients’ specific needs and improves the user-friendliness of the system [41,42]. A systematic review demonstrated the beneficial effects on patient involvement at all stages of the research process [43]. Hence, we pursued such an iterative approach, involving the user panel throughout the entire process of development and design.

#### 3.3.1. Participants

A total of ten users participated in the process. The users consisted of women between 33 and 75 years of age (mean, 50). They had all undergone various forms of cancer treatment (surgery, chemotherapy, and/or radiation) for different types of gynecological cancer, ranging from 1.5 to 11 years ago. They were all born in Norway. Three worked full-time, five worked part-time, partly with disability benefits, one received full disability benefits, and one was retired. Five women had a university degree (highest education), three graduated high school, and two had primary education only. Two were single, one was widowed, while seven lived with a partner. Eight women had children, while two of them lost their fertility due to cancer treatment. Half the women reported cancer-related fatigue and lymphedema, four reported occurrences of menopausal symptoms after treatment, reduced/lost femininity, sexual difficulties, and reduced libido, four reported radiation effects on the bowels, while three reported cancer-related neuropathies.

#### 3.3.2. Identification of Needs

Before we started the project, the first meeting was organized with the board of the Gynecological Cancer Association because of their lived experience of coping with cancer and their knowledge of other survivors’ experiences in the vicinity. They confirmed that their and others’ needs were not adequately met in the period following their diagnosis and treatment, including lack of psychosocial follow-up and a need for more holistic care. In particular, the women felt left to their own resources after discharge and with a sense that the hospital door was closed. A follow-up, with more focus on psychosocial needs, fear of recurrence, and sexuality, was emphasized as necessary by the women. It was concluded that there was an overall interest in a program that could improve and provide multidimensional support. Thus, we invited the users to participate in the process of developing our psychoeducational intervention.

In addition to the users, other stakeholders contributed to the process, including doctors, oncology nurses, a psychologist, a gestalt supervisor, a web designer, and an artist. Within the workshops, human–computer interaction (HCI) researchers contributed sketches and mockups of the user interface for the intervention. These early sketches greatly supported the discussions with the user panel. As the process matured, the prototype designs improved so that the Internet-delivered program would fit the intervention content and vice versa. Furthermore, we engaged an artist to produce illustrations to support the intervention content. The artist iterated the illustrations based on feedback from the user panel and other stakeholders.

#### 3.3.3. Workshops with the User Panel

The collaboration with the users was facilitated through eight workshops in the two-year development phase, from November 2017 until September 2019, where we generated ideas together, developed content, and evaluated texts, videos, images, and auditory exercises.

We prototyped and tested the content and usability in collaboration with the user panel (*n* = 5) in order to fully understand how to meet the needs, expectations, and feedback [29,44]. The users stressed throughout the workshop process that the information provided in the psychoeducational intervention should be easy to understand, closely related to a survivor’s experiences and that there should be other modalities for communicating the information in addition to text. They suggested that coaching or mindfulness could be ways to provide support, such as in dealing with fear of recurrence. The users also suggested that contact with health personnel throughout the program would be necessary for women to feel safe. Valuable feedback was given on the content and presentation style, imagery, and exercises. The content was discussed and reworked several times until all the parties were satisfied. Particularly important was the feedback on various parts of the intervention, for example, on the three written narratives presented at the beginning of the program, where due to their requests, complementary ways to communicate the stories were sought in view of the target users’ possibly reduced concentration span after treatment. This request resulted in the creation of three videos that featured illustrations created by the artist mentioned earlier to illustrate each character and her illness story narrated by a voice actor fitting the character. The panel gave valuable feedback regarding how they could identify with each narrative, expressed by one panelist like this: *“Oh, this story could have been about me!”* Another woman put it this way: *“I think the stories of the three women are very nice and trustworthy! I recognize and identify myself with the stories and I find many of my thoughts in the women’s descriptions.”* See [45] for a detailed study of the design and evaluation of these videos.

Throughout the iterative process, we collaborated to improve the content’s clarity, and the panel suggested which content to expand, such as sexual health after gynecological cancer. Regarding the mindfulness exercises, both texts and audio files were reviewed and supplemented with essential feedback, including the importance of guidance from a clear female voice using informal language: *“…a female voice…with the same body as me and who can immerse herself in the situation…because I think it’s easier for the patients to listen to someone of their own gender in relation to the situation they have been in.”* Another user representative expressed it this way: *“When you have had half of your femininity removed and you have to listen to a man’s voice…it wouldn’t have worked for me, to say the least.”* This feedback prompted further reworking and testing of the exercises during subsequent workshops until all the participants were satisfied.

The workshops also produced important feedback concerning the various styles of photographs and illustrations supporting the written content. The users associated several images with feelings and thoughts that we had not intended or were not helpful, again underlining the importance of the panel. For example, they associated a bent straw with reduced quality of life and impaired sexuality (a man’s impotence). Furthermore, they associated a classic sculpture of a headless body with a feeling of being half woman or living a “half-life” or having cancer–related fatigue. The users asked for realistic and concrete images, which could give hope, courage, and strength.

#### 3.3.4. Think Aloud Interviews with the Users

We ended the development phase with performing an evaluation following the think aloud protocol of the complete program, as suggested by Yardly et al. [29]. For this evaluation, our user panel included five additional users (*n* = 10). Each participant received printed copies of two modules by mail prior to the evaluation session. The program was evaluated in two-hour sessions, one participant at a time. In the evaluation, each participant was asked to browse through the two modules in the finished online platform. During their browsing, feedback and reflection on the content and the user interface were encouraged.

What stood out in the think aloud interviews, was the users’ identification with the program, especially the three fictive women who were presented in the first module and who returned as short stories and quotes throughout the whole program. All the ten participants identified themselves as cancer survivors and they all used the narratives as a starting point for sharing and discussing their own stories about living through cancer. They all approached the three fictional women in a personal way, which indicates that they experienced the narratives as credible and realistic. This was important as the purpose of the narratives and first-person perspective was that future users of the program could find support in recognizing and reflecting on their own experiences of illness, thus strengthening the individual woman’s self-competence and coping in an orientation to a new everyday life after cancer illness. The think aloud interviews gave valuable feedback on all parts of the web-based program, followed by minor adjustments in content and design.

**Figure 2:**
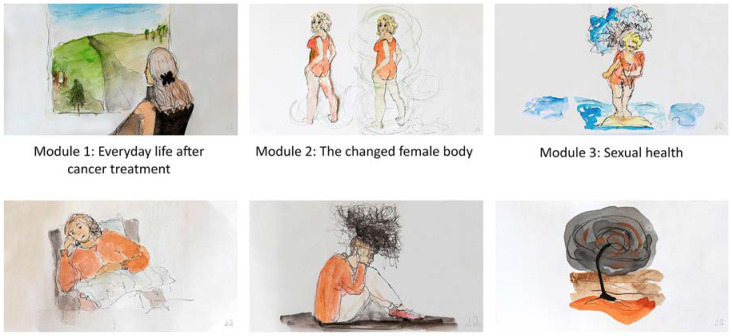
The program’s six modules.

The development phase resulted in a structured six-week Internet-delivered program with a new module opening every week for six consecutive weeks (Figure 2).

During the six modules, a dedicated nurse and a counselor are going to have a pre-scheduled telephone call with each participant on a weekly basis. The aim of the phone call is twofold: to support the women in their work with the modules (e.g., discuss situations and skills that the patients find difficult) and to collect qualitative data on the experiences with the intervention.

## 4. Discussion

Through a broad search on the existing evidence, choice of a theoretical framework, and collaboration with the user panel and other stakeholders, we developed a complex intervention targeting women’s needs after treatment for gynecological cancer. We suggest that the iterative process including collaboration with users over a two-year period and think aloud evaluations enhanced the intervention to be more relevant, recognizable, and user-friendly, an approach that is also recognized in other studies [41,42].

User involvement has been emphasized in health research over the last decades. The overall goal is to increase the research relevance and quality for the users. However, there are different approaches to user involvement [46]. From a consultation role, where the users are used to inform decision-making, via a more collaborating role involving a more active ongoing partnership where both parties share decisions, to a user-controlled research role in which the users have the power and the initiative to carry out the intervention [42,46]. In this study, the collaborative level of patient involvement was used, and a meeting with the users formed the start of the project.

Involving the users in the research project early in the process, working together in regular workshops over a period of over two years, as well as think aloud evaluations had a major impact on the development of the intervention’s content. The co-creative collaboration allowed for an iterative process to develop an appropriate Internet-delivered program through repeated feedback. The iterative process resulted in a more efficient and goal-directed way of producing content ensuring optimal alignment with the users’ needs [42]. The main motivation for the users was to help others in situations similar to the ones they had experienced, a finding in line with similar studies [42].

The gynecological cancer survivors’ felt lack of support as well as perceived lack of knowledge and understanding, were directly associated with a personal feeling of loss of control. In the case of breast cancer, [47,48] as well as gynecological cancer [14,49], studies have revealed that knowledge and understanding were fundamental in the process of becoming confident and familiar with an altered body in the aftermath. Lindwall and Bergbom [48] found that women regarded their body as “a stranger” and they had to regain familiarity with their altered bodies in the aftermath of treatment. This underscores the need for medical and psychosocial as well as an existential follow-up to best support the women in the period after treatment. In this intervention, this multidimensional support is given both in a more impersonal medical form and through women’s lived experiences. The three narratives express the recognizable psychosocial and existential aspects. Giving recognition to the existing emotions and the situation as well as providing guidance to deal with the challenges are also integrated in the program. Our intention is for this program to encourage and support more women to be active in their own recovery process, which hopefully will lead to a strengthened sense of self-competence and self-care after treatment for gynecological cancer.

As a result of gynecological cancer treatment, sexual health and relationships are particularly challenged [6,8,50,51,52]. This issue was reported as an unmet need by the users in current follow-up trajectories. Gynecological cancer affects body parts that are emotionally charged, associated with childbearing and sexuality, vulnerability, and taboos, frequently making it especially difficult to deal with. Most patients feel the urge to discuss sexuality and intimacy with health personnel but are reluctant to take the initiative. However, even though health personnel consider sexual health as an integral part of the overall treatment and care, it is often neglected in clinical care [53,54]. Lack of knowledge and training as well as one’s own bashfulness concerning a taboo issue are among the reasons suggested by health personnel for not addressing sexual health [54,55]. Therefore, an Internet-delivered program, in which sexual health and challenges are thematized, can be one way to educate and support patients and may help both patients and health personnel in overcoming their bashfulness.

Moreover, during the last decade, mindfulness and its effect on sexual health have received attention in health care in general [56,57,58] and with regard to the aftermath of gynecological cancer in particular [59]. A psychoeducational program that included components of mindful training for women with sexual problems after gynecological cancer showed how mindfulness was effective in improving the women’s sexual health and quality of life [59,60]. Consequently, the module on sexual health is quite extensive on the Internet-delivered program, and the three narratives are essential in sharing experiences related to sexual changes and challenges following cancer treatment. Further, the exercises for the module are aimed at self-support, self-care, and mindful practice, for both the individual and the couple. The focus is on sexuality as wellness and pleasure for the whole body.

Fear of cancer recurrence (FCR) is another major concern for patients and one of their most unmet needs. In addition, it is considered poorly addressed in the clinic [6,30,61,62,63,64,65]. Fear of recurrence is in part explained by the patients’ lack of knowledge of alarm symptoms and what to monitor in the aftermath of treatment. In a study by Olesen et al. [63], the knowledge was of importance to regain control over one’s own life. Inspired by this recent study, we therefore developed some “alarm symptom sheets” to assist the individual woman, in addition to describing signs and symptoms the patients may experience because of treatment and which are not alarming. Our hope is that this can lead to increased body knowledge and awareness of normality and thus more control over the perceived bodily signals and ultimately reduced stress levels.

However, fear of cancer recurrence is not eliminated by monitoring symptoms. A cancer diagnosis in itself affects the very existence of a human being and activates the fear of cancer recurrence [62,66,67]. New theoretical models on handling this fear focus not only on the complex content of the patient’s concerns, but include how individuals cognitively process information and their fear [68]. Several interventions have shown significant positive results on reduced FCR for cancer survivors [68,69]. These interventions combined, among others, components from mindfulness and acceptance and metacognitive perspectives [64,68]. Some other studies have also shown that mindfulness-based stress reduction improves patients’ adjustment to their disease [70]. Even though the current intervention is not an FCR intervention, it intends to prevent escalation of FCR by including medically relevant information, psychoeducation, and exercises. The main aim is that the women shall be able to accept the fear as a normal and helpful reaction but be aware that this fear can escalate and become dysfunctional.

When one’s life has been affected by cancer, it is changed in many ways. In this study, and as reported previously, patients tell about loneliness concerning their cancer experiences and the feeling that their loved ones have difficulties in understanding their concerns. Findings in the review made in support of the current study [6] showed that the women who had not been able to process the cancer experiences seemed to carry the greatest burden in the aftermath. This underscores the importance of integrating psychosocial and existential aspects of rehabilitation in the follow-up as offered in this intervention. Several studies that encompass awareness or mindful practice have also shown effect on mental health, such as reduced anxiety and depression, as well as decreased distress among cancer patients [71,72,73]. However, the main purpose of this program is to handle life following a cancer illness. In this perspective, we emphasize how to deal with life as it is through psychoeducation, acceptance, and self-care.

Usage of the UK Medical Research Council framework for developing complex interventions ensured the realization of a solid intervention. Using this systematic and structured approach also hopefully made the process more transparent for others.

Moreover, former gynecological cancer patients have been active throughout the whole development process. The users had different ages, diagnoses, treatment modalities, varying times after treatment, ages as well as sociodemographic backgrounds, and thus were a good cross-section of the gynecological cancer population. The users were encouraged to express their opinions during the workshops and think aloud interviews, to express divergent opinions and question the researchers’ ideas, something they clearly did. The users were very motivated to help and they often referred to how they would have reacted in the beginning after treatment, clearly identifying themselves with future users of the intervention.

The relationship between researchers and patients is traditionally asymmetrical, which can lead to user representatives’ knowledge and input to be unintentionally overruled [42]. We actively sought the users’ opinions and encouraged them to express such opinions, which they did. It is possible, however, that some experiences or disagreements were not expressed due to this unequal relationship.

## 5. Conclusions

Based on a broad evidence base, a solid theoretical foundation, and a prolonged collaboration with former gynecological cancer patients, we have developed an Internet-delivered psychoeducational intervention for women after treatment for gynecological cancer. With continuous feedback from the users, it was possible to make the intervention both relevant and attractive. The next step is to test the intervention in a clinical study.

## Data Availability

The data used and analyzed in this study will be promptly available to the publisher upon request.
